# Transcriptional Regulation of ING5 and its Suppressive Effects on Gastric Cancer

**DOI:** 10.3389/fonc.2022.918954

**Published:** 2022-06-07

**Authors:** Hua-chuan Zheng, Hang Xue, Xin Wu, Hai-lan Xu, En-hong Zhao, Zheng-guo Cui

**Affiliations:** ^1^Department of Oncology and Experimental Center, The Affiliated Hospital of Chengde Medical University, Chengde, China; ^2^Department of Pathology, Basic Medical College, Hebei North University, Zhangjiakou, China; ^3^Department of Gastrointestinal Surgery, The Affiliated Hospital of Chengde Medical University, Chengde, China; ^4^Department of Environmental Health, University of Fukui School of Medical Science, Fukui, Japan

**Keywords:** tumorigenesis, gastric cancer, ING5, transcriptional regulation, tumor suppressor

## Abstract

ING5 targets histone acetyltransferase or histone deacetylase complexes for local chromatin remodeling. Its transcriptional regulation and suppressive effects on gastric cancer remain elusive. Luciferase assay, EMSA, and ChIP were used to identify the cis-acting elements and trans-acting factors of the ING5 gene. We analyzed the effects of SAHA on the aggressive phenotypes of ING5 transfectants, and the effects of different ING5 mutants on aggressive phenotypes in SGC-7901 cells. Finally, we observed the effects of ING5 abrogation on gastric carcinogenesis. EMSA and ChIP showed that both SRF (−717 to −678 bp) and YY1 (−48 to 25bp) interacted with the promoter of *ING5* and up-regulated ING5 expression in gastric cancer *via* SRF-YY1-ING5-p53 complex formation. ING5, SRF, and YY1 were overexpressed in gastric cancer, (*P*<0.05), and associated with worse prognosis of gastric cancer patients (*P*<0.05). ING5 had positive relationships with SRF and YY1 expression in gastric cancer (*P*<0.05). SAHA treatment caused early arrest at S phase in ING5 transfectants of SGC-7901 (*P*<0.05), and either 0.5 or 1.0 μM SAHA enhanced their migration and invasion (*P*<0.05). The wild-type and mutant ING5 transfectants showed lower viability and invasion than the control (*P*<0.05) with low CDC25, VEGF, and MMP-9 expression. Gastric spontaneous adenocarcinoma was observed in Atp4b-cre; ING5^f/f^, Pdx1-cre; ING5^f/f^, and K19-cre; ING5^f/f^ mice. ING5 deletion increased the sensitivity of MNU-induced gastric carcinogenesis. ING5 mRNA might be a good marker of gastric carcinogenesis, and poor prognosis. ING5 expression was positively regulated by the interaction of SRF-YY1-ING5-p53 complex within the ING5 promoter from −50 bp upstream to the transcription start site. ING5 deletion might contribute to the tumorigenesis and histogenesis of gastric cancer.

## Introduction

The ING family is composed of ING1–5, functions as epigenetic readers of H3K4Me3 histone, and is involved in histone acetyltransferase (HAT) (1, 2). ING5 contains leucine zipper-like (LZL), novel conserved region (NCR), nuclear localization signal (NLS), and plant homeodomain (PHD) domains from its amino- to carboxyl-terminal. LZL is responsible for apoptotic induction, DNA repair, and chromatin remodeling. NCR might interact with HAT to assist chromatin remodeling and regulate transcription (2). ING5 mediates the acetylation of histone H3 *via* the HBO1 complex, and the acetylation of histone H4 by the MOZ/MORF complex. Two ING5 proteins form a homodimer *via* their amino-terminal domain and fold independently into a coiled-coil structure. ING5 contains a flexible and disordered NLS, responsible for binding to DNA. ING5 protein can also form heterodimers with ING4 protein (3).

ING5 is involved in two different HAT complexes: histone H4-ING5-HBO1-JADE and histone H3-ING5-MOZ-MORF-BRPF (4–8). Both H4-ING5-HBO1-JADE and MCM complexes contribute to DNA replication (9). The proteins acetylated by ING5 serve as transcription cofactors and chromatin remodelers in the nucleus, while are involved in metabolism in the cytosol. Interestingly, ING5 overexpression was found to promote p300 autoacetylation at lysine 1560, 1558, and 1555, and the acetylation of lysine 1647 and 1794 activated p300 HAT (10). In addition, ING5 was demonstrated to activate p21 expression and acetylate p53 protein (11). ING5 was identified as an interaction partner and inhibitor of Cyclin A1 that bound to Cyclin A1/CDK2 (12). Moreover, ING5 facilitated Tip60-increased acetylation of p53 at lysine 120 and induced apoptosis by up-regulating the transcription of Bax and GADD45 (13).

The low nuclear expression of ING5 and its nucleocytoplasmic translocation were observed in the tumorigenesis of breast cancer, head and neck squamous cell carcinoma, colorectal cancer, and gastric cancer, and closely linked to invasion and metastasis (14–17). Although high ING5 expression was detected in gastric, colorectal, breast, and lung cancers (15–18), ING5 was found to be expressed at a low level in osteosarcoma, ovarian cancer, prostate cancer, and hepatocellular carcinoma (19–22), which might be possibly due to the different normal control tissues, tissue specificity of ING5 expression and the distinct infiltration contents of interstitial cells, and subsquently result in their different biological functions. ING5 overexpression was also shown to inhibit proliferation, migration, invasion, and tumor growth, or promote apoptosis and lipogeneisis in prostate cancer cells by suppressing Akt and activating p53 (22), in lung cancer cells by β-catenin phosphorylation at ser33/37 (23) and the EGFR/PI3K/Akt and IL-6/STAT3 pathway (24), in neuroblastoma cells by histone acetylation (25), in colorectal cancer cells by sthe PI3K/Akt pathway (26), in osteosarcoma cells by the Smad pathway (20), in esophageal squamous carcinoma cells through the Akt/NF-κB/MMP-9 pathway (27), in ovarian (19) and breast cancer cells *via* PI3K/Akt/NF-κB (17, 28, 29), or in glioma cells (30), and gastric cancer cells (31) *via* the PI3K/Akt or β-catenin/TCF-4 pathway. ING5 overexpression was seen in chemosensitive bladder cancer cells, and ING5 silencing enhanced the chemoresistance (32), opposite to our findings (17, 18, 30, 31). ING5 expression inhibited the effects of hepatocyte growth factor on the proliferation, invasion, and epithelial-to-mesenchymal transition (EMT) of thyroid cancer cells *via* the c-Met/PI3K/Akt pathway (32). In addition, ING5 can block the stimulating effects of miR-200b/200a/429 on proliferation and clone formation of ovarian cancer cells (33). Moreover, ING5 also promotes stemness and self-renewal, and prevented lineage differentiation in glioblastoma cells *via* Ca^2+^ and follicle-stimulating hormone (34). To clarify the molecular mechanisms of ING5 overexpression and its possible roles of ING5 in gastric carcinogenesis, we here explored the promoter sequences and transcriptional factors of ING5 gene, and analyzed the clinicopathological and prognostic significances of ING5 and trans-acting factors in gastric cancer. The synergistic effects of ING5 and SAHA were also determined in gastric cancer cells. Finally, we clarified the effects of ING5’s domains on the phenotypes of gastric cancer cells and ING5 knockout on gastric carcinogenesis using conditional knockout (KO) mice.

## Materials and Methods

### Cell Culture and Transfection

The gastric cancer cell lines (SGC-7901 and AGS) and HEK293 cells were from the Cell Bank of the Chinese Academy of Sciences. They were cultured in DMEM supplemented with 10% fetal bovine serum (FBS) at 37°C. SGC-7901 cells were transfected with FLAG-tagged wild-type (WT) and mutant MUT ING5 with their vector as mock. To screen the promoter activity, we constructed pGL3-2000WT and MUT, 1500WT and MUT, 1000WT and MUT, 800WT and MUT, 650WT and MUT, 400WT and MUT, and 100WT and MUT (Jiangsu ProbeGene Biotechnology), which were transfected into HEK293 cells. SRF siRNA (sc-36563) and YY1 siRNA (sc-36863) were purchased from Santa Cruz and used for their expression silencing in SGC-7901 and AGS.

### Proliferation

CCK-8 was used to detect cell viability. Briefly, 2500 cells/well were grown on a 96-well plate until adherence. Ten microliters of CCK-8 solution was then dispensed into each well of the plate. After incubation for 3 h, the absorbance was measured at 450 nm.

### PI (Propidium Iodide) and IdU (Iododeoxyuridine)/CldU (Chlorodeoxyuridine) Staining

For the cell cycle analysis and the detection of early and late DNA synthesis, DNA contents were determined by PI staining, and DNA synthesis was visualized by the double staining of CldU and IdU as previously described (31).

### Apoptosis Assay by Flow Cytometry

For apoptotic analysis, flow cytometry was carried out with PI and FITC-labeled Annexin V (Keygen) double staining to determine phosphatidylserine externalization as described in the manufacturer’s recommended protocol.

### Wound Healing Assay

For wound healing assay, cells were grown in a six-well plate until they reached about 80% confluence. Then, the cells were scraped with a pipette tip, rinsed with PBS, and cultured in FBS-free DMEM medium. Cells were photographed at 24h, 48h, and 72 h. The healing area was measured and calculated using Image J software.

### Transwell Assay

For the migration assay, 250,000 cells were resuspended in FBS-free DMEM and dispensed in a Matrigel-coated membrane insert on the top of the chamber (Corning). DMEM with 7% FBS was present as a chemo-attractant in the lower compartment. After incubation for 1 day, we scrubbed the top cells on the membrane, rinsed with PBS, fixed in 100% ethanol, and stained the membrane with crystal violet. For the invasion assay, the procedures were similar to the migration assay, excluding the use of the matrigel-coated insert.

### Immunofluorescence

Both AGS and SGC-7901 were seeded on glass coverslips, fixed with 4% formaldehyde for 10 min, and permeabilized with 0.5% Triton X-100 for 10 min. After washed with PBS, cells were incubated with the combination of anti-rabbit SRF (CST), YY1 (CST) or ING5 (Proteintech) antibody with anti-mouse p53 (Proteintech) antibody for 1h. The cells were then incubated with Alexa Fluor 488 (green) anti-mouse and Alexa Fluor 568 (red) anti-rabbit IgG (Invitrogen) for 1h. Nuclei were stained with DAPI (Keygen) at 37°C.

### Dual-Luciferase Reporter Assay

We performed luciferase assay using Dual-Luciferase^®^Reporter Assay System (Promega, USA). Briefly, HEK293 cells were cultured in a 12-well plate for 36 h after the addition of 2.0 µg pGLs and 20 µg pRL-TK. We removed the growth medium from the cells and rinsed them twice in PBS. 1× cell lysis reagent was dispensed into each well and incubated for 5 min. Then, we scraped the cells and transferred them into EP tubes for pelleting. The supernatant was transferred into a new tube, and mixed with 20 µl of cell extract with 100 µl of Luciferase Assay Reagent. The reaction was placed in a luminometer and the light produced for 10 s was measured.

### Electrophoretic Mobility Shift Assay

The biotin-labeled (experiment), label-free (cold competition), and mutant double-stranded DNA oligonucleotides containing Sp-1, PPAR-γ1, WT-1, SRF, YY1, PAX-5 or CTCF binding site (mutation cold competition) are shown in **Supplementary Table 1**. We dissolved the forward and reverse oligonucleotides to a concentration of 10 μM, and annealed and diluted them to form double-stranded DNA probe at 50 nM. The transcription factors (Sp-1, PPAR-γ1, WT-1, SRF, YY1, PAX-5, or CTCF) were prepared by his-tagged pET32T and purified by Ni-IDA. We prepared 5% non-denaturing polyacrylamide gels and subjected the DNA–protein (5–10μg) mixtures to electrophoresis. Then, we carefully transferred the gel to a nylon membrane, which was subsequently cross-linked. After incubation with streptavidin-HRP conjugate, the membranes were visualized using ECL reagents.

### Chromatin Immunoprecipitation (ChIP)

ChIP assay was carried out using Magna ChIP™ G kit (Millipore, USA). The primer sequences were targeted to the ING5 promoter at approximately 2 kb upstream. For the immunoprecipitation (IP) of anti-SRF antibody, we used P1F (5’-gcatgcatcttacggcacac-3’) and P1R (5’-gccacctctcgaggcagg-3’). For IP of anti-YY1 antibody, we used P2F (5’-cgcgcgactcatg aatagtg-3’) and P2R (5’-agtgctccaagtacatggcg-3’). Anti-polymerase II was employed as a positive control and IgG as a negative control. DNA was amplified in 20 µl mixture and separated in 1% agarose gel.

### Co-Imunoprecipitation (Co-IP)

More than 1mg protein was pre-cleared with 50 μl protein A-Sepharose beads for 60 min with gentle rotation, and incubated with 5 μg primary antibody (**Supplementary Table 2**) overnight on a rotator. After that, 100 μl protein A sepharose beads were added and rotated at 4°C for overnight. The samples were centrifugated to remove non-specific binding proteins. The beads were washed 5 times with 1% NP40 lysis buffer. The pellet was eluted by 50 μl 2× SDS sample buffer and heated at 100°C for 10 min. The samples were pelleted and the supernatant was prepared for western blot.

### Animals

Mice were bred three per plastic cage and supplied with corn chips, standard high-nutrition food, and water. We maintained them under specific-pathogen-free condition with 12-h light/dark cycling. The Committee on Animal Experimentation of Affiliated Hospital of Chengde Medical University approved all of our experiments, and mice care was in accordance with the committee guidelines. We developed targeted abrogation of ING5 by mating ING5 mutant (prepared by Shanghai Biomodel) with Atp4b (parietal-cell-specific)-cre and Capn8-cre (pit-cell-specific) mice (kindly provided by Prof. Xiao Yang), PGC-cre (gastric chief cells, prepared by Shanghai Biomodel), K19-cre (stem-like cells) (35), and Pdx1-cre (Jax Lab) mice. To promote gastric carcinogenesis, we orally administered N-nitroso-N-methylurea (MNU, 240 mg/l) to Pdx1- cre/ING5^f/f^ and K19-cre/ING5^f/f^. Finally, we euthanized the mice by CO_2_ asphyxiation and removed the stomach, fixed it in 4% formaldehyde, and prepared into tissue blocks.

### Polymerase Chain Reaction

DNA was extracted from the mouse tail and stomach using proteinase K/phenol/chloroform. We carried out PCR targeting ING5 and cre, used Hotstart polymerase (Takara) to confirm the genotype, and applied PCR of stomach DNA to verify the conditional knockout of ING5. The primers for ING5 were as follows: P1: 5’-TCCTCTCTGGTTCAGGCAGA-3’, P2:5’ -CTAAATGAGTACACTTACAC-3’, P3:5’-AGAGCAGTCAGTGCTCCCAA-3’, P4:5’-AGAGCAGTCAGTGCTCCCAA-3’and P5: 5’-AATGAGCAGAAGAGGACGAG-3’.

### Reverse Transcription-Polymerase Chain Reaction

Total RNA was extracted from cancer cells using Trizol (Takara) and subjected to cDNA synthesis using AMV reverse transcriptase and random primer (Takara). As described elsewhere (31), RT-PCR was performed to amplify ING5 using Hotstart polymerase. The primers for ING5 were Forward, 5’-GAGGACATCAGAGGAAGACACAC -3’ and Reverse, P2:5’-CACTCAATTGGACAGTCTGGATT-3’(198bp). The primers for SRF were Forward, 5’-CACAACAGACCAGAGAATGAGTG-3’ and Reverse, 5’-GTAGAGGTGCTAGGTGCTGTTTG -3’(171bp). The primers for YY1 were Forward, 5’-AGAAGAGCGGCAAGAAGAGTTAC-3’ and Reverse, 5’-CAATGACCC CTTCATTGACC-3’(192bp). The primers for GAPDH were Forward, 5’-TGGAAGATGGTGATGGGATT-3’ and Reverse, 5’-TGGAAGATGGTGATGGGATT-3’ (135bp).

### Western Blot

Protein was extracted in RIPA lysis buffer, measured using a Bio-Rad protein assay kit, separated in 10% SDS-PAGE gel, and transferred to a PVDF membrane (Amersham). After that, the membrane was blocked overnight in 5% milk, and exposed to primary antibody (**Supplementary Table 2**) and IgG conjugated to horseradish peroxidase (DAKO), as reported previously (31). Bands were visualized by Azure C300 Biosystem using ECL-Plus detection reagents.

### *In situ* PCR

Ten-micrometer-thick sections were deparaffinized and subjected to *in situ* PCR, as previously described (35). The primers for exon 4 of ING5 were P6, 5’-ACCATAACCCACCACAGC-3’ and P7, 5’-TTACACCAGTCCGTCCCT-3’ (156bp).

### Bioinformatics Analysis

The mRNA expression of ING5, SRF, and YY1 was analyzed using Oncomine (www.oncomine.org), Xiantao platform (http://xiantao.love), or UALCAN (http://ualcan.path.uab.edu/). We analyzed the correlations of ING5 mRNA expression with clinicopathological characteristics of the patients with gastric cancer, and analyzed the relationships between ING5, SRF, and YY1 expression using TCGA database. Their prognostic significance was also analyzed using Kaplan–Meier Plotter (https://kmplot.com/) and the Xiantao platform.

### Statistical Analysis

Either Student’s *t*-test or One-way ANOVA was performed to compare the means, and both Kaplan–Meier curve analysis and Cox’s proportional risk model were used to analyze survival. *P<*0.05 was considered as statistically significant. SPSS 10.0 software was employed to analyze all data.

## Results

### Promoter Activity and Trans-Acting Factors of *ING5* Gene

**Figure 1:**
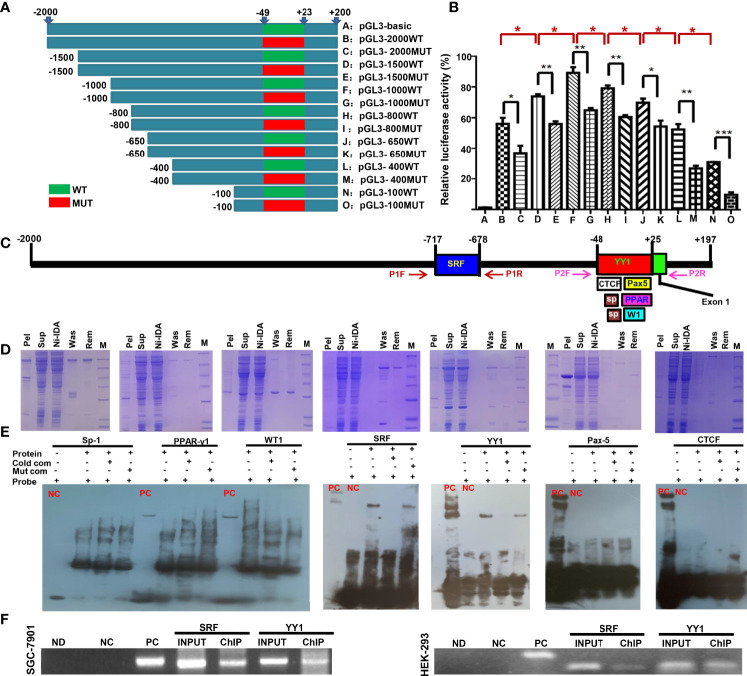
The promoter activity and trans-acting factors of *ING5* gene Different upstream wild-type (WT) and mutant (MUT) DNA fragments of ING5 were inserted into the pGL-3 basic vector, as shown schematically **(A)**. These plasmids and pRL-TK were co-transfected into HEK293 cells and the cell lysates were subjected to luciferase reporter assay **(B)**. We also predicted trans-acting factors that bind to the promoter of ING5 **(C)**. Among them, His-tagged Sp-1 (Sp), WT-1 (W1), PPAR-γ1 (PPAR), SRF, YY1, CTCF, and Pax5 expression plasmids were constructed using pET-28 vector, induced by IPTG, and purified by Ni-IDA **(D)**. In EMSA, the recombinant proteins were incubated with probes and subjected to electrophoresis **(E)**. To confirm the EMSA results, we carried out ChIP in SGC-7901 and HEK293 cells using either anti-SRF or anti-YY1 antibody **(F)**. M, marker; pel, pellet after sonication; sup, supernatant after sonication; Ni-IDA, supernatant throughout Ni-IDA column; Was, washed target protein; Rem, remnant protein in column; NC, negative control; PC, positive control; cold com, cold competitor; Mut com, mutant competitor; ChIP, chromatin immunoprecipitation; Ctr, control; ND, no DNA; NC in ChIP, negative control using normal mouse IgG ChIP; PC in ChIP, positive control using anti-Pol II ChIP; INPUT, 2% input.

The eukaryotic promoter database and Neural Network Promoter Prediction were employed to predict the promoter sequence of *ING5*. As **Figure 1A** indicates, we designed different upstream DNA fragments of *ING5* gene, which were inserted into the pGL-3 basic vector. Additionally, we randomly mutated the promoter sequence (-48~+23) in 5’-Taacatggacaatcggacattgcgatccttgcggaaacttggttaggactaggccaacactcctgggaccatca-3’ (**Figure 1A**). The luciferase reporter assays showed lower promoter activity in all mutants than for the corresponding WT DNA fragments (**Figure 1B**, *P*<0.05). The promoter activity gradually increased from −2000 bp, −1500 bp to −1000 bp (*P*<0.05), while it decreased from −800 bp, −650 bp, −400 bp to −100 bp (**Figure 1B**, *P*<0.05).

Subsequently, we also used AliBaba2.1, PROMO, and Genecards to predict the trans-acting factors of *ING5* promoter and selected Sp1 (−44 to −30 bp; −32 to −20), PPAR-γ1 (−24 to 25 bp), WT1 (−10 to −1 bp), SRF (−717 to −678 bp), YY1 (−48 to 25 bp), Pax-5 (−1 to 25 bp), and CTCF (−48 to 0 bp, **Figure 1C**). Their recombinant GST-tagged proteins were prepared and purified using the pET28a system (**Figure 1D**). We designed biotin-labeled, unlabeled protein, and unlabeled mutant probes. EMSA showed that only SRF and YY1 could bind to the promoter (−50 to 0 bp) of the ING5 gene (**Figure 1E**), which was confirmed by ChIP (**Figure 1F**).

**Figure 2:**
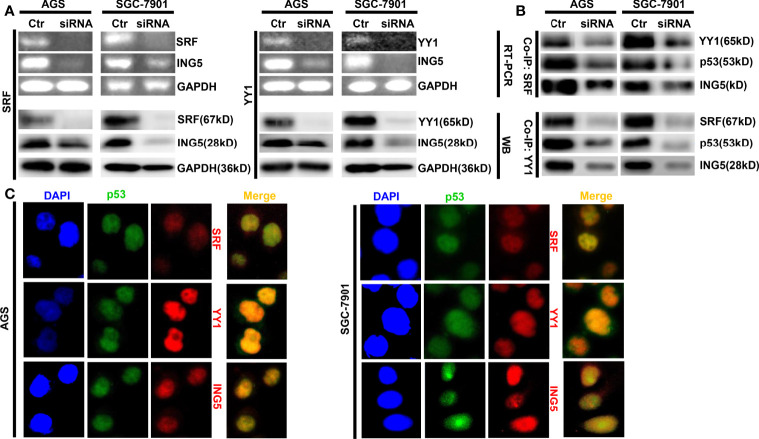
The promoting effects of both SRF and YY1 proteins on ING5 expression The results of RT-PCR and Western blot revealed the successful silencing of SRF and YY1 expression in AGS and SGC-7901 cells, which showed low *ING5* mRNA and protein expression **(A)**. Co-IP was performed to explore whether SRF bind to YY1, p53 and ING5 or YY1 to SRF, p53 and ING5 in both gastric cancer cells, treated with SFR or YY1 siRNA **(B)**. Double immunofluorescence was carried out to observe the co-localization of YY1, SRF or ING5, and p53 in gastric cancer cells **(C)**. Ctr, control; RT-PCR, reverse-transcriptional polymerase chain reaction; WB, western blot; Co-IP, co-immunoprecipitation.

In SGC-7901 and AGS cells, we silenced the expression of either SRF or YY1 at both mRNA and protein levels and found that ING5 mRNA and protein were hypoexpressed in either SRF or YY1 siRNA transfectants (**Figure 2A**). In both cells, SRF might bind to YY1, p53 and ING5, which was weakened after SRF or YY1 knockdown (**Figure 2B**). Either SFR or YY1 protein was co-localized with p53 or ING5 in gastric cancer cells, evidenced by double immunofluorescence (**Figure 2C**).

### Clinicopathological Significance of *ING5*, *SRF*, and *YY1* mRNA Expression in Gastric Cancer

**Figure 3:**
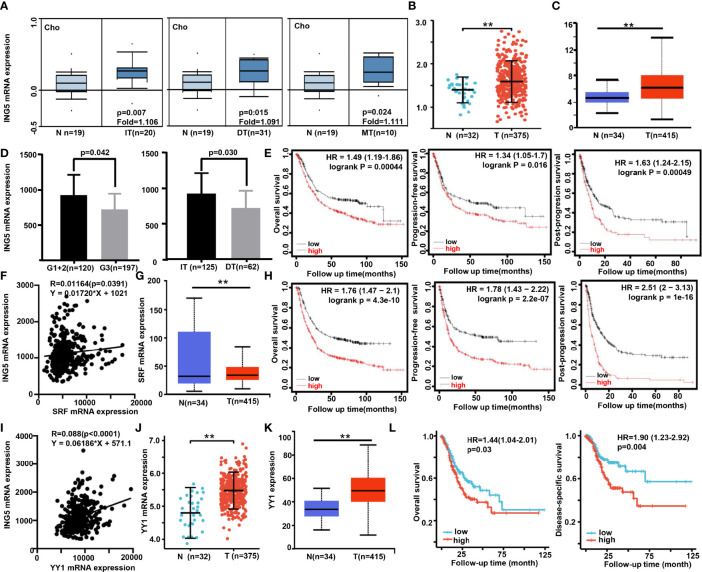
The clinicopathological significance of *ING5*, *SRF* and *YY1* mRNA expression in gastric cancer Oncomine **(A)**, Xiantao **(B)**, and/or UALCAN **(C)** datasets were employed to analyze *ING5* mRNA expression in gastric cancer, and the correlations of its expression with pathological parameters of cancers were analyzed using TCGA **(D)**. Kaplan–Meier curves were used to analyze the relationships of *ING5* mRNA expression with overall (OS), progression-free (PFS), and post-progression (PPS) survival, according to Kaplan-Meier plotter **(E)**. The relationship between SFR and *ING5* mRNA expression was analyzed using TCGA data **(F)**. SRF mRNA expression was explored in gastric cancer using UALCAN **(G)**. The prognostic significance of SRF mRNA expression was investigated using Kaplan-Meier plotter **(H)**. We also investigated the relationship between *YY1* and *ING5* mRNA expression using TCGA data **(I)**. *YY1* mRNA expression was explored in gastric cancer using Xiantao **(J)** and UALCAN **(K)**. The prognostic significance of *SRF* mRNA expression was studied using Xiantao platform **(L)**. N, normal mucosa; T, tumor; IT, intestinal type; DT, diffuse type; MT, mixed-type; HR, hazard ratio. ; **p<0.01.

Next, we found that *ING5* mRNA expression was higher in gastric cancer than in normal tissues, even when stratified into intestinal-, diffuse-, and mixed-type carcinoma using Cho’s datasets (**Figure 3A**, *P*<0.05). According to Xiantao (**Figure 3B**) and UALCAN (**Figure 3C**) databases, it was increased in gastric cancer, compared with the level in normal mucosa (*P*<0.05). In TCGA data, it was negatively correlated with histological grading and differentiation (**Figure 3D**, *P*<0.05). Meanwhile, Kaplan-Meier plotter showed that *ING5* mRNA expression was negatively correlated with overall survival (OS), progression-free (PFS) survival, and post-progression survival (PPS) rates of cancer patients (**Figure 3E**, *P*<0.05). There was also a positive correlation between *ING5* and *SRF* mRNA expression in gastric cancer (*P*<0.05, **Figure 3F**). In addition, UALCAN showed higher *SRF* mRNA expression in gastric cancer than in normal mucosa (**Figure 3G**, *P*<0.05). *SRF* mRNA expression was negatively associated with OS, PFS, and PPS of gastric cancer patients (**Figure 3H**, *P*<0.05). In TCGA data, there was a positive correlation between *ING5* and *YY1* mRNA expression in gastric cancer (**Figure 3I**, *P*<0.05). Both Xiantao (**Figure 3J**) and UALCAN (**Figure 3K**) datasets showed higher *SRF* mRNA expression in gastric cancer than in normal mucosa (*P*<0.05). Finally, *SRF* mRNA expression was negatively associated with OS and disease-free survival (DSS) of gastric cancer patients, according to the Xiantao platform (**Figure 3L**, *P*<0.05).

As shown in **Supplementary Table 3**, univariate and multivariate survival analyses demonstrated that T staging, N staging, M staging, pathological staging, and *YY1* mRNA expression were closely linked to unfavorable OS and DSS survival of gastric cancer patients (*P*<0.05). However, only *YY1* mRNA expression was an independent factor for worse OS and DSS (*P*<0.05).

### Anti-Tumor Effects of SAHA on ING5-Overexpressing Gastric Cancer Cells

**Figure 4:**
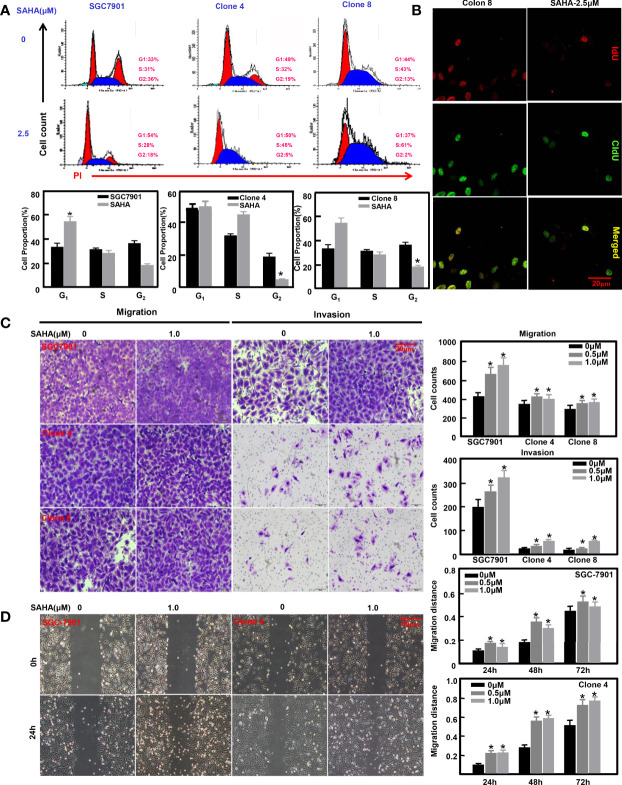
The anti-tumor effects of SAHA on ING5-overexpressing gastric cancer cells SGC-7901 and its ING5 transfectants (Clones 4 and 8) were treated with SAHA at 0.5 and 1.0 μM. After exposure, they were subjected to PI staining and flow cytometry **(A)**. Among them, clone 8 was stained using IdU and CIdU to differentiate the early and late phases of DNA synthesis **(B)**. Transwell **(C)** and wound healing **(D)** assays were employed to analyze the effects of SAHA on migration and invasion of these cells. Note: *, compared with 0μm, p<0.05.

Previously, we overexpressed ING5 in SGC-7901 cells (31). SAHA treatment caused G_1_ arrest of SGC-7901 cells (*P*<0.05), but S-phase arrest in ING5 transfectants (**Figure 4A**, *P*<0.05). Additionally, IdU and CIdU integration indicated that ING5 transfectants showed early arrest in S phase (**Figure 4B**). We also exposed SGC-7901 cells and their ING5 transfectants to SAHA, and found that either 0.5 or 1.0 μM SAHA increased their migration and invasion, as shown by the results of transwell (**Figure 4C**, *P*<0.05) and wound healing assays (**Figure 4D**, *P*<0.05).

### Effects of Different ING5 Domains on Biological Phenotypes of Gastric Cancer Cells

**Figure 5:**
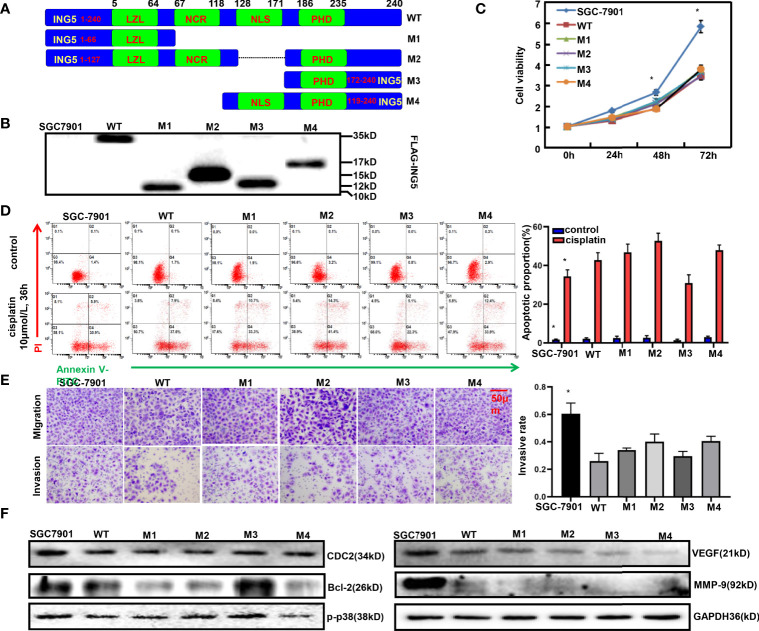
The effects of ING5 domains on phenotypes and related proteins of gastric cancer cells FLAG-tagged wild-type (WT) and mutant (M1–M4) ING5 fragments were inserted into pcDNA3.1 vector **(A)**. These WT and mutant ING5-expressing plasmids were transfected into SGC-7901 cells by Western blot **(B)**, and all transfectants were subjected to the examination of cell viability **(C)**. After treatment with cisplatin (10 μmol/L, 36 h), we also employed PI-Annexin V-FITC staining to examine apoptosis **(D)**. Transwell assay was utilized to observe migration and invasion **(E)**. Finally, phenotype-related proteins were screened by western blot **(F)**. Note: *, compared with mutant, p<0.05.

To clarify the effects of different ING5 domains on the phenotypes, we constructed four mutants as shown in **Figure 5A**. After transfection with wild-type (WT) or mutant ING5-expressing plasmid, SGC-7901 cells were shown to overexpress WT and truncated ING5 by Western blot using anti-FLAG antibody (**Figure 5B**). These transfectants showed lower viability than the maternal cells (*P*<0.05, **Figure 5C**). Except for the M3 ING5 transfectant, there was a higher level of early apoptosis in the ING5 transfectants of SGC-7901, even upon treatment with cisplatin (**Figure 5D**, *P*<0.05). Additionally, different ING5 overexpression could suppress migration and invasion (**Figure 5E**, *P*<0.05). Western blot revealed that CDC-2, VEGF, and MMP-9 expression was higher in control cells than in WT and mutant ING5 transfectants (**Figure 5F**). The same findings were made for Bcl-2 and p-p38, but not for the M3 ING5 transfectant (**Figure 5F**).

### Effects of ING5 on Gastric Carcinogenesis

**Figure 6:**
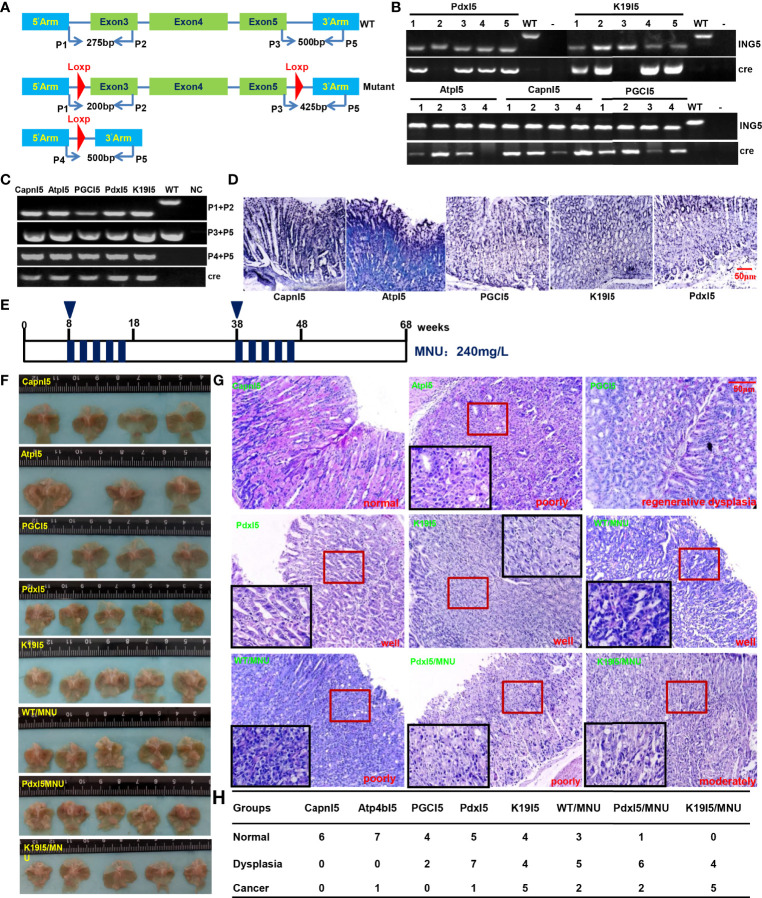
The effects of conditional *ING5* knockout on gastric carcinogenesis PCR primers were designed **(A)** and used for PCR of tail **(B)** DNA. We performed tube **(C)** and *in situ* **(D)** PCR amplification targeting mutant and deleted *ING5* using the stomach samples of Capn8-cre/ING5^f/f^ (CapnI5), Atp4b-cre/ING5^f/f^ (AtpI5), PGC-cre/ING^f/f^ (PGCI5), Pdx1-cre/ING5^f/f^ (PdxI5), and K19-cre/ING^f/f^ (K19I5) and. MNU was orally administered into these wild-type and conditional knockout mice in accordance with the schedule **(E)**. The stomach of wild-type (WT) and knockout (KO) mice was grossly **(F)** and histologically **(G)** observed with or without exposure to MNU (240 mg/L) until 68 weeks. The histological findings on the gastric lesions are summarized in **(H)**.P, primer; NC, negative control.

We designed primers to confirm the genotype using tail DNA and target abrogation of ING5 using gastric mucosal DNA (**Figure 6A**). Tube and *in situ* PCR results (**Figures 6B–D**) confirmed the successful targeted deletion of ING5 in gastric epithelial cells in these conditional knockout ING5 mice. Among them, Pdx1-cre^+/−^;ING5^f/f^ and K19-cre^+/−^;ING5^f/f^ were orally administered by a chemical gastric carcinogen (MNU) as scheduled (**Figure 6E**). Grossly, no remarkably protruding lesions were observed in Capn8-cre^+/−^;ING5^f/f^ and PGC-cre^+/−^;ING5^f/f^ mice, but were seen in other gastric target knockout mice, and MNU-treated WT and KO mice (**Figure 6F**). Histologically, there was normal gastric epithelium in Capn8-cre^+/−^;ING5^f/f^ mice and regenerative dysplasia in PGC-cre^+/−^;ING5^f/f^ mice (**Figure 6G**). However, poorly, moderately, or well-differentiated adenocarcinoma was observed in the other conditional KO mice, and MNU-treated WT and KO mice (**Figure 6G**). As summarized in **Figure 6H**, we found that spontaneous gastric dysplasia and cancer were more frequently seen in K19-cre^+/−^;ING5^f/f^ and Pdx1-cre^+/−^;ING5^f/f^ mice than in the other target KO mice. After the exposure to MNU, chemically-induced gastric dysplasia and cancer were more common in K19-cre^+/−^;ING5^f/f^ and Pdx1-cre^+/−^;ING5^f/f^ mice than in WT mice.

## Discussion

Although the nucleocytoplasmic translocation of ING5 protein appeared from gastric mucosa, dysplasia to cancer (16), ING5 was observed to be upregulated in gastric cancer in comparison with the level in paired mucosa at both mRNA and protein levels (16, 31). To clarify why ING5 is up-regulated in gastric cancer, we predicted the promoter sequence and screened its activity. Both EPD and BDGP predicted the presence of a promoter of *ING5* from −50 bp upstream to the transcription start site, which was evidenced by mutation and reporter gene assays. Additionally, luciferase assay demonstrated that there was a suppressor between −2000 and −1000 bp, and an enhancer between −800 and −100bp. Although we predicted that 7 such transcription factors bind to ING5 promoter, namely, SP1, PPAR-γ1, WT1, SRF, YY1, Pax-5, and CTCF, EMSA and ChIP assays showed that only SRF and YY1 might interact with the promoter (−50 to 0 bp) of *ING5*.

According to the bioinformatics findings, the expression of *ING5*, *SRF*, and *YY1* mRNA was higher in gastric cancer than in normal mucosa, and negatively associated with favorable prognosis of gastric cancer patients. We also found positive correlations of *ING5* mRNA expression with *SRF* and *YY1* mRNA expression and a negative effect of either *SRF* or *YY1* knockdown on *ING5* mRNA expression. Reportedly, SRF expression was frequently elevated in a panel of metastatic gastric cancer cells and tissues, and high expression of SRF was significantly associated with a more aggressive phenotype and poor prognosis in gastric cancer patients (36) or facilitating crosstalk between myofibroblasts and cancer cells in an SDF1-CXCR4-dependent manner (37). YY1 directly binds to CCDC43 and ADRM1 gene promoters, leading to their overexpression, and subsequently the aggressiveness of gastric cancer (38). In addition, YY1 expression was increased in gastric cancer tissues (39), in line with our results. In gastric cancer cells, *YY1* knockdown inhibited Wnt/β-catenin, JNK/MAPK, ERK/MAPK, ER, and HIF-1α signaling pathways (40). Here, we found that SRF bound to YY1, p53 and ING5 in gastric cancer cells. Either *SRF* or *YY1* silencing ameliorated the SRF-YY1-ING5-p53 complex formation. SRF, YY1, or ING5, and p53 were co-localized in the nuclei of gastric cancer cells. Taking these findings in combination, we speculated that both SRF and YY1 interacted with the promoter of *ING5* and up-regulate its expression in gastric cancer cells *via* the formation of SRF-YY1-ING5-p53 complex.

ING5 overexpression has been reported to inhibit aggressive phenotypes of gastric cancer cells by β-catenin, NF-κB, and Akt pathways (31). Here, we treated ING5-overexpressing transfectants of SGC-7901, and found that SAHA induced the early arrest in S phase of ING5 transfectants, and promoted their migration and invasion at a low concentration (<1.0μM). Previously, SAHA treatment was demonstrated to suppress proliferation, tumor growth, glucose metabolism, or the formation of lamellipodia, and induced G_2_ arrest and apoptosis in glioma, ovarian, and gastric cancer cells (41–43). In addition, SAHA increased cell migration and invasion at a low concentration, as shown by transwell and wound healing assays (43), in line with the present findings for ING5 transfectants. ING5 and acetylated histones H3 and H4 were recruited to the promoters of c-myc, Nanog, Cyclin D1, p21, and p27 for complex formation, thereby regulating the mRNA expression of downstream genes. Taking these findings together, ING5 might be used as a target for the anti-tumor effect of SAHA in various cancer cells (25). However, efforts should be made to prevent the effects of SAHA on promoting migration and invasion regardless of the ING5 expression level.

We also constructed ING5 variants according to the protein domains and clarified the effects of these domains on the phenotypes of gastric cancer cells. These transfectants were found to have lower viability and invasion than the maternal cells, consistent with the cdc-2, VEGF, and MMP-9 expression. All WT and mutant ING5 have either the LZL or PHD domain, which might be responsible for the inhibition of proliferation. Cyclin B1-Cdk1 is involved in the early events of mitosis, and CDC25B activates the Cyclin-dependent kinase CDC2 for entry into mitosis (44). MMP-9 degrades the extracellular matrix for cancer cell invasion, stimulates angiogenesis, and increases VEGF release for cancer cell proliferation and angiogenesis (45). The low expression of CDC2, VEGF, and MMP-9 might account for the inhibitory effects of WT and mutant ING5 on the proliferation and invasion of gastric cancer. Additionally, a higher level of early apoptosis was observed in WT and mutant ING5 transfectants than in SGC-7901 cells, even upon treatment with cisplatin, except for the phenomenon in the M3 mutant, in agreement with the findings on Bcl-2 and p-p38 expression. M3 mutant only contained the PHD domain, indicating that this domain is not necessary for apoptotic induction. Reportedly, Bcl-2 can interact with Bax on the mitochondrial membrane to suppress apoptosis because Bax opens voltage-dependent anion channels for apoptosis (46). P38 MAPK is activated by phosphorylation and then upregulates Bax/Bcl-2 for mitochondrial apoptosis (47). Therefore, we speculated that WT and mutant ING5 might inhibit apoptosis *via* Bcl-2 and p-p38, which is not closely linked to the PHD domain of ING5.

Here, we for the first time abrogated ING5 in gastric parietal, stem-like, pit, chief, and pdx1-positive cells and found poorly, moderately, or well-differentiated gastric carcinoma in Atp4b-cre;ING5^f/f^, Pdx1-cre;ING5^f/f^, and K19-cre;ING5^f/f^ mice, suggesting that ING5 deletion in parietal, stem-like, and Pdx1-positive cells of gastric epithelium might play an important role in the histogenesis of gastric cancer. Additionally, ING5 KO in stem-like and Pdx1-positive cells increased the susceptibility to chemically-induced gastric carcinogenesis. Taking these findings together, we concluded that ING5 functions as a tumor suppressor in gastric cancer.

In summary, ING5 expression was shown to be transcriptionally regulated by the interaction of SRF and YY1 with the ING5 promoter −50 bp upstream of the transcription start site, and subsequently up-regulated in gastric cancer *via* the formation of SRF-YY1-ING5-p53 complex. ING5 might be used as a target for the anti-tumor effect of SAHA in gastric cancer cells if its promoting effects on migration and invasion are avoided or ameliorated. ING5 deletion in parietal, stem-like, and Pdx1-positive cells of gastric epithelium might contribute to the histogenesis of gastric cancer, and increase the susceptibility to chemically induced gastric carcinogenesis. Therefore, we can confidently employed ING5 as a biomarker for aggressiveness and prognosis of gastric cancer and a target of gene therapy for gastric cancer patients.

## Data Availability Statement

The original contributions presented in the study are included in the article/**Supplementary Material**. Further inquiries can be directed to the corresponding author.

## Ethics Statement

The animal study was reviewed and approved by The committee of The Affiliated Hospital of Chengde Medical University. Written informed consent was obtained from the owners for the participation of their animals in this study.

## Author Contributions

Conception and design: H-CZ. Provision of study materials or patients: E-HZ and XW. Collection and assembly of data: HX and H-LX. Data analysis and interpretation: H-CZ and Z-GC. Manuscript writing: All authors. All authors contributed to the article and approved the submitted version.

## Funding

This study was supported by Award for Liaoning Distinguished Professor, Natural Science Foundation of Hebei Province (21377772D) and National Natural Scientific Foundation of China (81672700).

## Conflict of Interest

The authors declare that the research was conducted in the absence of any commercial or financial relationships that could be construed as a potential conflict of interest.

## Publisher’s Note

All claims expressed in this article are solely those of the authors and do not necessarily represent those of their affiliated organizations, or those of the publisher, the editors and the reviewers. Any product that may be evaluated in this article, or claim that may be made by its manufacturer, is not guaranteed or endorsed by the publisher.
