# Supplementary material for: Transcriptional Regulation of ING5 and its Suppressive Effects on Gastric Cancer
Source: Front Oncol. 2022 Jun 7;12:918954. doi: 10.3389/fonc.2022.918954 (PMC9209732; doi:10.3389/fonc.2022.918954)
Supplement: Supplementary file 1 [file Table_1.docx]

**Supplementary table 1** The probes used in electrophoretic mobility shift assay

| **TF** | **Probe** | **DNA sequence** |
| --- | --- | --- |
| **Sp1** | Biotin-labeled | 5’-cgctggcaccgccccgcccccgcctcccgcggcaccgcccgcccgcgcaGACCCCGAGCGcggccgcggacgaa-3’-biotin |
|  | Cold competition | 5’-cgctggcaccgccccgcccccgcctcccgcggcaccgcccgcccgcgcaGACCCCGAGCGcggccgcgga cgaa-3’ |
|  | Mutant | 5’-cgctggcGccgcgccGgGtcgcccccccctccgccaccccggccgcgcgaccggCgCcAacCcGaggcgacgaa-3’ |
| **WT1** | Biotin-labeled | 5’-cgctggcaccgccccgcccccgcctcccgcggcaccgcccgcccgcgcaGACCCCGAGCGcggccgcggacgaa-3’- biotin |
|  | Cold competition | 5’-cgctggcaccgccccgcccccgcctcccgcggcaccgcccgcccgcgcaGACCCCGAGCGcggccgcggac gaa-3’ |
|  | Mutant | 5’-cgctggcaccgcagatatcaatcctcccgcggcaccgcttataacaataGACCCCGAGCGcggccgcggacgaa-3’ |
| **PPAR-γ1** | Biotin-labeled | 5’-cgctggcaccgccccgcccccgcctcccgcggcaccgcccgcccgcgcaGACCCCGAGCGcggccgcggac gaa-3’-biotin |
|  | Cold competition | 5’- cgctggcaccgccccgcccccgcctcccgcggcac cgcccgcccgcgcaGACCCCGAGCGcggccgcggac gaa-3’ |
|  | Mutant | 5’-ccCCggcGccgcgccGgGtcgcccccccctccgccaccccggccccaggaccggCgCcAacCcGaggccaA ggg-3’ |
| **SRF** | Biotin-labeled | 5’-ttattaactatatcatactgccgcacgtgccttttcggccaaggcatcca-3’ -biotin |
|  | Cold competition | 5’-ttattaactatatcatactgccgcacgtgccttttcggccaaggcatcca-3’ |
|  | Mutant | 5’-aataattgatatagtatgacggcgtgcacggaaaagccggttccgtaggt-3’ |
| **YY1** | Biotin-labeled | 5’-cgctggcaccgccccgcccccgcctcccgcggcaccgcccgcccgcgcaGACCCCGAGCGcggccgcggacgaa-3’ -biotin |
|  | Cold competition | 5’-cgctggcaccgccccgcccccgcctcccgcggcaccgcccgcccgcgcaGACCCCGAGCGcggccgcggacgaa-3’ |
|  | Mutant | 5’-gcgaccgtggcggggcgggggcggagggcgccgtggcgggcgggcgcgtctggggctcgcgccggcgcctgctt-3’ |
| **CRCF** | Biotin-labeled | 5’-cgctggcaccgccccgcccccgcctcccgcggcaccgcccgcccgcgcaGACCCCGAGCGcggccgcggacgaa-3’ -biotin |
|  | Cold competition | 5’-cgctggcaccgccccgcccccgcctcccgcggcaccgcccgcccgcgcaGACCCCGAGCGcggccgcggacgaa-3’ |
|  | Mutant | 5’-gcgaccgtggcggggcgggggcggagggcgccgtggcgggcgggcgcgtctggggctcgcgccggcgcctgctt-3’ |
| **Pax-5** | Biotin-labeled | 5’-cgctggcaccgccccgcccccgcctcccgcggcaccgcccgcccgcgcaGACCCCGAGCGcggccgcggacgaa-3’-biotin |
|  | Cold competition | 5’-cgctggcaccgccccgcccccgcctcccgcggcaccgcccgcccgcgcaGACCCCGAGCGcggccgcggacgaa-3’ |
|  | Mutant | 5’-gcgaccgtggcggggcgggggcggagggcgccgtggcgggcgggcgcgtctggggctcgcgccggcgcctgctt-3’ |

Note: TF, transcription factor.

**Supplementary table 2** The primary antibodies used in the present study

| **Num** | | **Antibody** | **Species** | **Dilution** | **Company** | |
| --- | --- | --- | --- | --- | --- | --- |
| 1 | SRF(D71A9) | | Rabbit | 1:1000 | | CST |
| 2 | YY1(D5D9Z) | | Rabbit | 1:1000 | | CST |
| 3 | ING5(ab259904) | | Animal free | 1:2000 | | Proteintech |
| 4 | GAPDH(ab8245) | | Mouse | 1:2000 | | Proteintech |
| 5 | P53(1C12) | | Mouse | 1:1000 | | CST |
| 6 | DYKDDDDK Tag(D6W5B) | | Rabbit | 1:1000 | | CST |
| 7 | Cdc2 p34(SC-54) | | Mouse | 1:500 | | Santa Cruz |
| 8  9  10  11 | BCL-2(D17C4)  Phospho-p38MARK(Thr180/Tyr182)(D3F9)  VEGF(SC7269)  MMP-9(SC-393859) | | Rabbit  Rabbit  Mouse  Mouse | 1:1000  1:1000  1:500  1:500 | | CST  CST  Santa Cruz  Santa Cruz |

**Supplementary table 3** The survival analysis in gastric cancer patients

| **Characteristics** | **Overall survival** | | | | **Disease-specific survival** | | | |
| --- | --- | --- | --- | --- | --- | --- | --- | --- |
|  | **Univariate** | | **Multivariate** | | **Univariate** | | **Multivariate** | |
|  | **HR (95%CI)** | ***P*** | **HR (95%CI)** | ***P*** | **HR (95%CI)** | ***P*** | **HR (95%CI)** | ***P*** |
| Age  (>65 vs. <=65) | 1.620 (1.154-2.276) | **0.005** | 1.856 (1.272-2.707) | **0.001** | 1.211 (0.797-1.840) | 0.371 |  |  |
| Gender  (Female vs. Male) | 1.267 (0.891-1.804) | 0.188 |  |  | 1.573 (0.985-2.514) | 0.058 | 1.569 (0.959-2.567) | 0.073 |
| T stage  (T3-4 vs. T1-2) | 1.719 (1.131-2.612) | **0.011** | 1.062 (0.626-1.800) | 0.824 | 2.089 (1.192-3.660) | **0.010** | 1.520 (0.768-3.007) | 0.229 |
| N stage  (N1-3 vs. N0) | 1.925 (1.264-2.931) | **0.002** | 1.249 (0.694-2.249) | 0.459 | 1.807 (1.075-3.036) | **0.025** | 1.014 (0.491-2.092) | 0.971 |
| M stage  (M1 vs. M0) | 2.254 (1.295-3.924) | **0.004** | 1.885 (0.977-3.637) | 0.059 | 2.438 (1.221-4.870) | **0.012** | 1.658 (0.784-3.508) | 0.186 |
| Pathologic stage (III- IV vs. I-II) | 1.947 (1.358-2.793) | **<0.001** | 1.421 (0.810-2.495) | 0.221 | 2.146 (1.352-3.404) | **0.001** | 1.513 (0.757-3.026) | 0.242 |
| Histologic grade  (G3 vs. G1-2) | 1.353 (0.957-1.914) | 0.087 | 1.404 (0.958-2.058) | 0.082 | 1.338 (0.862-2.078) | 0.194 |  |  |
| ING5 expression  (High vs. Low) | 0.934 (0.673-1.297) | 0.685 |  |  | 1.194 (0.783-1.819) | 0.410 |  |  |
| SRF expression  (High vs. Low) | 1.004 (0.723-1.394) | 0.981 |  |  | 1.159 (0.759-1.770) | 0.495 |  |  |
| YY1 expression  (High vs. Low) | 1.443 (1.037-2.008) | **0.030** | 1.515 (1.053-2.179) | **0.025** | 1.898 (1.232-2.923) | **0.004** | 1.837 (1.161-2.908) | **0.009** |

Note: HR, hazard ratio; CI, confidence interval.
